# Lichen planus pigmentosus inversus induced by the Koebner phenomenon following laser hair removal

**DOI:** 10.1016/j.jdcr.2026.01.029

**Published:** 2026-01-27

**Authors:** Nada Hussain Alghamdi, Alzahraa Bader AlAhmed, Meshail Mansour Alsaud, Alaa Khalaf AlHowaish, Abdulaziz Mohammed Alotaibi, Nadia Abdullah AlAudah

**Affiliations:** a Department of Dermatology, SCFHS- Ministry of Health, Dammam, Saudi Arabia; b Department of Dermatology, Ministry of Health (MOH), Dammam, Saudi Arabia; c Department of Dermatology, Saudi Board Certified, Ministry of Health, Dammam, Saudi Arabia; d Department of Dermatology, German Board Certified, Ministry of Health, Dammam, Saudi Arabia; e Department of Pathology, Saudi Board Certified, Ministry of Health, Dammam, Saudi Arabia

**Keywords:** case report, Koebnerization phenomenon, laser hair removal, lichen planus

## Introduction

Lichen planus pigmentosus inversus (LPPI) is an uncommon form of lichen planus.^1^ The condition primarily affects the axillae, sub-mammary region, abdomen, groin, popliteal fossa, and lumbar spine. Although the exact cause of LPPI remains uncertain, it is postulated that mechanical stress, such as from tight clothing, may play a role in triggering the Koebner phenomenon, where lesions develop in areas of trauma or irritation.^2^ Herein, we present a rare case of an uncommon occurrence of LPPI in a 30-year-old female, highlighting its induction through the Koebnerization phenomenon (KP) following laser hair removal.

## Case presentation

A 30-year-old Saudi woman with Fitzpatrick skin phototype III, and no significant medical or dermatological history presented to our dermatology clinic with gradually developing hyperpigmented lesions in both axillae over 1 year. The axillary lesions began as hyperpigmented, mildly pruritic macules that gradually became darker in color over time. This was followed by hyperpigmented patches on the left inner thigh and mons pubis, which also progressively darkened over the following months. No preceding erythema, vesiculation, pain, or oozing was reported. Systems review was unremarkable. When asked about potential triggers, she reported undergoing full-body Alexandrite (755 nm) and Nd:YAG (1064 nm) laser hair removal 2 weeks prior to the appearance of the lesion.

She had undergone multiple sessions of laser hair removal, with a 1-month interval between each session. She denied prior episodes of similar pigmentation or use of topical treatment. There was no family history of notable pigmentary disorders.

**Fig 1:**
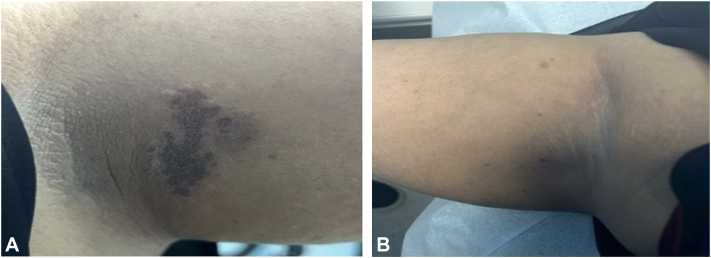
Lichen planus pigmentosus inversus. **A,** a well-defined, brown-colored patch with violaceous hue surrounded by skin-colored papules and an ill-defined hyperpigmented patch involving the left axilla. **B,** multiple scattered ill-defined hyperpigmented macules in the right axilla.

**Fig 2:**
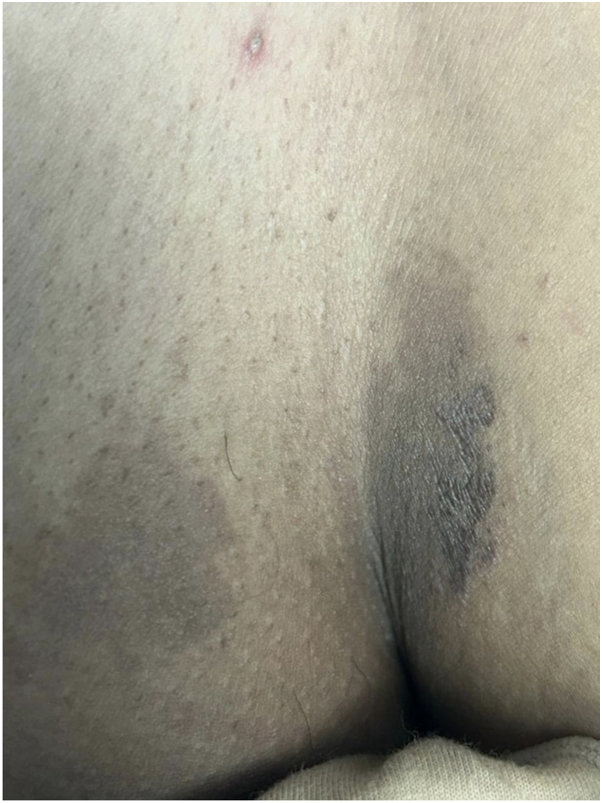
Lichen planus pigmentosus inversus. A well-demarcated hyperpigmented patch and multiple macules.

A dermatological examination revealed a well-defined hyperpigmented brown-colored patch with violaceous hue with 1- to 2-mm violaceous satellite papules (Fig 1, *A*) and multiple scattered ill-defined hyperpigmented macules on the right axilla (Fig 1, *B*). There were multiple hyperpigmented patches on the mons pubis and left inner thigh, with a variable color gradient, surrounded by 1-2 mm violaceous papules on the inner thigh (Fig 2). No erythema, scaling, or induration was observed. No abnormalities were noticed on the palms, soles, scalp, nails, or mucous membranes.

**Fig 3:**
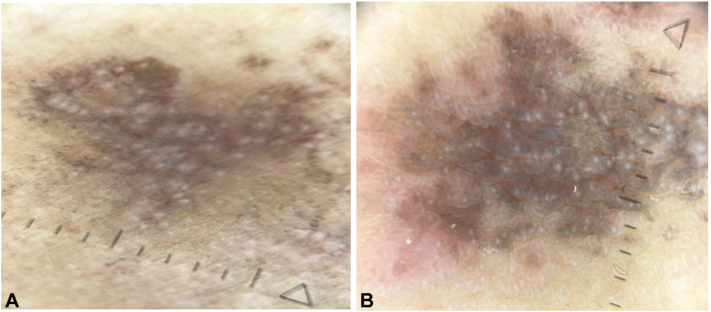
Dermatoscopy of the left inner thigh **(A)** and axilla **(B)** showing superficial lichenoid inflammatory infiltrates with focal vacuolar degeneration and Civatte bodies, consistent with lichen planus pigmentosus inversus.

Using dermatoscopy as a diagnostic aid, we observed diffuse violaceous-brown pigmentation with fine gray dots and granules and absence of Wickham striae of lesions on the axilla and inner thighs (Fig 3). Our initial clinical impression for these lesions included LPPI, post-inflammatory hyperpigmentation and erythema dyschromicum perstans.

**Fig 4:**
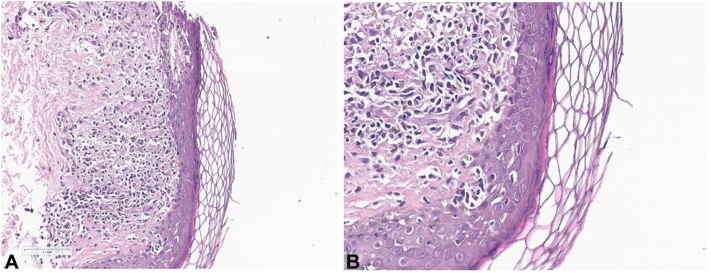
Histopathology showing superficial lichenoid inflammatory infiltrates with vacuolar degeneration of the basal layer and Civatte bodies, consistent with lichen planus pigmentosus inversus.

The patient underwent a 3-mm punch biopsy of the left axilla. Histopathology showed superficial lichenoid inflammatory infiltrate—a band-like arrangement of lymphocytes and histiocytes (Fig 4, *A* and *B*). Focal vacuolar degeneration of the basal cell layer with civatte bodies was also seen. These findings, along with the clinical presentation, were compatible with LPPI. The patient was initially treated with mometasone cream twice daily along with hydroquinone at bed time.

At the 1-month follow-up, there was scant improvement of symptoms. A T.R.U.E patch test demonstrated mild allergic reactions to quaternium and p-phenylenediamine, and a severe reaction to Disperse Blue 106. The complete blood count with differential, renal and liver function tests, and hepatitis B and C serology were unremarkable.

## Discussion

The term LPPI was introduced by Pock et al in 2001, following the report of 7 cases of a rare variant of lichen planus pigmentosus predominantly affecting the intertriginous areas.^3^ Clinically, LPPI commonly presents as small, lenticular, hyperpigmented macules, typically with little to no itching. These macules are most frequently found in the intertriginous areas, with the axillae being affected in 90% of the reported cases. Other common sites include the neck, groin, and the area behind the knees.^1^ Although the exact etiology remains unclear, several etiological factors have been suggested, including T lymphocyte-mediated cytotoxic activity against basal keratinocytes.^4^ Associations with hepatitis C and triggers such as chemicals, metals, and friction from tight-fitting clothing have been reported.^5^ While KP is known to play a role in the occurrence, progression, persistence, and relapse of lichen planus, there are no studies directly linking LPPI with laser hair removal.^6^ However, iatrogenic factors, such as the use of laser hair removal are recognized as triggers for KP in LPPI. Our case report is unique due to the rare presentation of LPPI, with the potential involvement of KP contributing to the onset of the condition.

## Conclusion

This case report presents an uncommon occurrence of LPPI in a 30-year-old female, highlighting its induction through KP following laser hair removal. We believe this case will serve as an educational resource for clinicians, helping them recognize and understand this rare diagnosis, its clinical presentation, and triggers.

### Declaration of generative AI and AI-assisted technologies in the writing process

Artificial intelligence tools were used only for grammar and language editing. No AI tool was used for data analysis, content generation, or interpretation.

## Conflicts of interest

None disclosed.

## References

[1] Roster K., Tarawneh O.H., Zufall A. (2023). Dermatoscopic rainbow pattern in lichen planus pigmentosus inversus in a middle-aged African American man. JAAD Case Rep.

[2] Alsoleman M.M., Alkheder A., Fathallah I., Alsodi Z. (2025). Persistent lichen planus pigmentosus inversus in a 45-year-old woman: a decade-long clinical journey and therapeutic challenges in anuncommon dermatological variant. Ann Med Surg (Lond).

[3] Pock L., Jelínková L., Drlík L. (2001). Lichen planus pigmentosus–inversus. J Eur Acad Dermatol Venereol.

[4] Bennàssar A., Mas A., Julià M., Iranzo P., Ferrando J. (2009). Annular plaques in the skin folds: 4 cases of lichen planus pigmentosus-inversus. Actas Dermosifiliogr.

[5] Gavazzoni F.M., Patta F., Nobile C., Girolami I. (2025). Lichen planus pigmentosus inversus: case report and systematic review. Dermatol Rep.

[6] Zhang X., Lei L., Jiang L. (2023). Characteristics and pathogenesis of Koebner phenomenon. Exp Dermatol.

